# Association of systemic immune-inflammation index with all-cause mortality in lung cancer patients undergoing radiotherapy: a retrospective cohort study

**DOI:** 10.3389/fonc.2026.1806392

**Published:** 2026-05-04

**Authors:** Jianwei Yang, Zhihui Liu, Maoze Zhang

**Affiliations:** Department of Radiotherapy, Sunshine Union Hospital, Weifang, Shandong, China

**Keywords:** all-cause mortality, lung cancer, prognostic biomarker, radiotherapy, systemic immune-inflammation index

## Abstract

**Background:**

The systemic immune-inflammation index (SII) has emerged as a widely studied inflammatory biomarker reflecting the balance between host inflammatory and immune status, although evidence in lung cancer patients undergoing radiotherapy remains limited. This study aimed to investigate the association between baseline SII and all-cause mortality in lung cancer patients undergoing radiotherapy.

**Methods:**

A retrospective cohort study was conducted including 489 lung cancer patients who received radiotherapy from January 2022 to January 2025. Baseline SII was calculated as platelet count × neutrophil count/lymphocyte count. Patients were categorized into three tertiles based on SII values: T1 (low SII, ≤480.69, n = 159), T2 (moderate SII, 480.69–857.56, n = 171), and T3 (high SII, >857.56, n = 159). The primary endpoint was all-cause mortality, with follow-up until death or September 2025. Cox proportional hazards models, subgroup analyses, Kaplan–Meier survival analysis, receiver operating characteristic (ROC) curve analysis, and restricted cubic spline (RCS) analysis were employed to assess the relationship between SII and mortality risk.

**Results:**

During a median follow-up of 19.9 months, 269 patients (55.0%) died from all causes. Kaplan–Meier analysis showed significantly poorer survival in patients with high SII (T3) compared with those in the low SII group (T1, P < 0.001). In multivariable Cox models, SII remained an independent predictor of all-cause mortality. When analyzed as tertiles, the fully adjusted hazard ratios (HRs) were 2.583 (95% confidence interval [CI]: 1.630–4.095) for T2 and 4.475 (95% CI: 2.841–7.047) for T3 compared with T1 (P for trend < 0.001). Standardized SII also consistently predicted higher mortality risk (HR = 1.222, 95% CI: 1.108–1.347, P < 0.001). Subgroup analyses confirmed stronger associations in patients aged ≤ 60 years and in both sexes. ROC curve analysis demonstrated that SII had strong discriminative power for predicting all-cause mortality, with an area under the curve (AUC) of 0.831 (95% CI: 0.794–0.865, P < 0.001). RCS analysis revealed a significant non-linear relationship between SII and all-cause mortality (P-overall < 0.001; P-nonlinear < 0.001).

**Conclusion:**

High baseline SII predicts higher all-cause mortality in lung cancer patients receiving radiotherapy, indicating its value as a simple and cost-effective prognostic biomarker.

## 1 Introduction

Lung cancer remains one of the most prevalent and lethal malignancies worldwide, posing a major threat to human health (1). Although advances in surgery, radiotherapy, chemotherapy, and targeted immunotherapy have led to certain survival benefits, the overall prognosis is still unsatisfactory (2). For patients who are not candidates for surgery, radiotherapy plays a crucial role in achieving local control and prolonging survival (3). However, even among those receiving standardized radiotherapy, survival outcomes vary widely. This variation underscores the need for reliable biomarkers to better predict prognosis and to guide individualized treatment strategies (4).

Accumulating evidence suggests that systemic inflammation and immune status are deeply involved in tumor initiation, progression, and outcomes (5). In the tumor microenvironment, inflammatory responses can drive tumor growth, angiogenesis, and metastasis, while simultaneously impairing anti-tumor immunity, thereby accelerating disease progression (6). Consequently, indices that reflect the balance between inflammation and immunity have gained increasing attention. Conventional hematologic parameters—such as neutrophil, lymphocyte, and platelet counts—have been used to derive composite scores, including the neutrophil-to-lymphocyte ratio (NLR), platelet-to-lymphocyte ratio (PLR), and the systemic immune-inflammation index (SII) (7–9). Among these, SII incorporates platelet, neutrophil, and lymphocyte counts into a single metric, providing an integrated reflection of the interplay between pro-tumor inflammation and anti-tumor immunity (8). Compared with single-parameter markers, SII offers a more comprehensive assessment of host immune-inflammatory status and has shown greater prognostic relevance (10). Previous studies have demonstrated that SII is significantly associated with survival outcomes in hepatocellular carcinoma, gastric cancer, and colorectal cancer, and may also predict treatment response (8, 11, 12). Although SII has been widely investigated as an inflammatory index in various cancers, evidence regarding its prognostic role in lung cancer—particularly among patients undergoing radiotherapy—remains relatively limited (13, 14). Given that radiotherapy not only directly eradicates tumor cells but also exerts complex effects on the immune system—such as enhancing tumor antigen release and immune activation, while at times inducing immunosuppression—investigating the prognostic significance of SII in this context is of particular scientific and clinical value (15, 16).

Based on this rationale, the present retrospective cohort study aims to evaluate the association between SII levels and all-cause mortality in lung cancer patients treated with radiotherapy. We hypothesize that elevated SII reflects heightened inflammatory activity and impaired immune defense, and is therefore linked to worse outcomes. The objectives of this study are threefold: (1) to determine whether SII is an independent predictor of all-cause mortality in this patient population; (2) to assess its utility in prognostic stratification among radiotherapy recipients; and (3) to provide clinicians with a simple and cost-effective prognostic tool that may facilitate precision treatment, optimize follow-up strategies, and lay the groundwork for future prospective investigations.

## 2 Methods

### 2.1 Study population and design

This was a single-center retrospective cohort study conducted at Sunshine Union Hospital. The study population consisted of patients diagnosed with lung cancer who underwent radiotherapy at the hospital between January 2022 and January 2025. A total of 503 cases were initially identified, and clinical as well as laboratory information was retrieved from the electronic medical record system. Patient eligibility was determined according to predefined criteria. Inclusion criteria were: (1) histological or cytological confirmation of lung cancer; (2) receipt of definitive or palliative radiotherapy during hospitalization; and (3) availability of complete baseline hematological data before the initiation of radiotherapy. Exclusion criteria were: (1) history of another malignant tumor; (2) evidence of active infection, autoimmune disease, or hematologic disorder at baseline that could affect the SII; (3) radiotherapy performed at an outside institution; (4) presence of severe hepatic failure or uremia; and (5) loss to follow-up. After applying these criteria, 489 patients were deemed eligible and included in the final analysis. The study was conducted in accordance with the ethical principles of the Declaration of Helsinki and received approval from the Ethics Committee of Sunshine Union Hospital (2025031422). All participants (or their legally authorized representatives) provided written informed consent prior to enrollment.

### 2.2 Definition and grouping of SII

The SII was calculated as platelet count (×10^9^/L) × neutrophil count (×10^9^/L)/lymphocyte count (×10^9^/L). All hematological parameters were derived from routine peripheral blood tests performed before the initiation of radiotherapy. According to the tertile distribution of SII levels, patients were divided into three groups: T1 (SII ≤ 480.69), T2 (480.69 < SII ≤ 857.56), and T3 (SII > 857.56).

### 2.3 Follow-up and outcome assessment

All patients were followed from the date of hospital discharge until death or September 2025, whichever occurred first. Follow-up information was obtained primarily from post-discharge outpatient and emergency department records, subsequent hospitalization data, and was supplemented by telephone interviews to ensure completeness and accuracy of outcome ascertainment. The primary endpoint of this study was all-cause mortality, defined as death from any cause. In addition, overall survival (OS) was recorded, which was defined as the interval from hospital discharge to death or the last follow-up. For patients who were still alive at the end of follow-up, survival time was censored at the date of last contact.

### 2.4 Collection and assessment of other data

The study also collected demographic, clinical, and laboratory information. Demographic variables included age, sex, body mass index (BMI), smoking history, and blood pressure levels (systolic and diastolic), which were used to evaluate the patients’ general health status and baseline metabolic condition. Clinical comorbidities included the presence of hypertension and diabetes. Tumor characteristics encompassed histological type (categorized as adenocarcinoma or other types of lung cancer), stage (III or IV), and site of metastasis (intrapulmonary or distant). Laboratory parameters were obtained from routine hematology and biochemical examinations conducted before radiotherapy. These included: (1) hematologic indices such as white blood cell count, neutrophil count, lymphocyte count, monocyte count, hemoglobin, and platelet count; (2) liver function markers including alanine aminotransferase (ALT), aspartate aminotransferase (AST), total bilirubin, and serum albumin; (3) renal function indicators including blood urea nitrogen (BUN), serum creatinine, and uric acid; and (4) metabolic parameters including fasting blood glucose (FBG) and lipid profile [triglycerides, total cholesterol, high-density lipoprotein cholesterol (HDL-C), and low-density lipoprotein cholesterol (LDL-C)]. All data were extracted from the hospital’s electronic medical record system by two independent investigators, and cross-checked to ensure completeness and accuracy.

### 2.5 Statistical analysis

All continuous variables were tested for normality using the Shapiro-Wilk test. Since none of the variables followed a normal distribution, they were summarized as medians with interquartile ranges (M [P25, P75]), and group comparisons were performed with the Mann-Whitney U test or Kruskal-Wallis test. Categorical variables were presented as counts and percentages, and differences between groups were assessed using the chi-square test or Fisher’s exact test where appropriate. Survival outcomes were analyzed with the Kaplan-Meier method, and differences across SII tertiles were evaluated using the log-rank test. Associations between SII and all-cause mortality were estimated with Cox proportional hazards regression models, reporting hazard ratios (HRs) and 95% confidence intervals (CIs). Univariable Cox analysis was first performed, and variables with P < 0.1 were included in multivariable models. Model 1 was adjusted for age and sex; Model 2 was additionally adjusted for hypertension, diabetes, histological type (adenocarcinoma vs. other), and tumor stage; Model 3 further included systolic blood pressure (SBP), diastolic blood pressure (DBP), creatinine, FBG, triglycerides, and LDL-C. Subgroup analyses were conducted according to age and sex, and adjusted HRs with 95% CIs were calculated to assess the associations within each subgroup using multivariable Cox proportional hazards models. The predictive ability of SII for all-cause mortality was assessed using receiver operating characteristic (ROC) curves, and the area under the curve (AUC) with 95% CIs was calculated. To explore potential non-linear associations between SII and mortality risk, restricted cubic spline (RCS) models were fitted. All statistical tests were two-sided, and a P value < 0.05 was considered statistically significant. Analyses were conducted using SPSS software (version 28.0, IBM Corp., Armonk, NY, USA) and R software (version 4.4.3, R Foundation for Statistical Computing, Vienna, Austria).

## 3 Results

### 3.1 Baseline characteristics

**Table 1** summarized the baseline characteristics of the 489 patients with lung cancer. The median age was 62 years, with 44.6% male and 55.4% female. During follow-up, 269 patients died from all causes, while 220 remained alive. Compared with survivors, patients in the death group had higher proportions of hypertension and diabetes, a greater frequency of distant metastasis, and lower BMI and serum albumin levels, accompanied by higher SBP and DBP, elevated counts of white blood cells, neutrophils, monocytes, and platelets, reduced levels of BUN, uric acid, and triglycerides, as well as higher HDL-C, lower LDL-C, and markedly increased SII and standardized SII (all P < 0.05).

**Table 1:** Patient demographics and baseline characteristics.

Characteristics	All-cause mortality			P value
	Overall	No	Yes	
N	489	220	269	
Age, years	62.00 (54.00, 71.00)	60.00 (55.00, 73.00)	63.00 (53.00, 68.00)	0.082
Sex, n (%)				0.725
Male	218 (44.6%)	100 (45.5%)	118 (43.9%)	
Female	271 (55.4%)	120 (54.5%)	151 (56.1%)	
Smoking, n (%)	404 (82.6%)	177 (80.5%)	227 (84.4%)	0.254
Hypertension, n (%)	124 (25.4%)	43 (19.5%)	81 (30.1%)	0.008
Diabetes, n (%)	42 (8.6%)	10 (4.5%)	32 (11.9%)	0.004
Adenocarcinoma, n (%)	444 (90.8%)	204 (92.7%)	240 (89.2%)	0.182
Staging, n (%)				0.132
Stage III	33 (6.7%)	19 (8.6%)	14 (5.2%)	
Stage IV	456 (93.3%)	201 (91.4%)	255 (94.8%)	
Site of metastasis, n (%)				0.011
Intrapulmonary metastasis	285 (58.3%)	142 (64.5%)	143 (53.2%)	
Distant metastasis	204 (41.7%)	78 (35.5%)	126 (46.8%)	
Body mass index, kg/m^2^	22.64 (20.62, 25.65)	22.64 (21.09, 25.95)	22.27 (19.92, 25.59)	0.012
Systolic blood pressure, mmHg	129.00 (116.00, 139.00)	127.00 (105.00, 139.00)	130.00 (120.00, 140.00)	0.002
Diastolic blood pressure, mmHg	79.00 (70.00, 87.00)	78.00 (69.00, 84.00)	80.00 (74.00, 89.00)	<0.001
White blood cell count, x10^9^/L	5.51 (4.47, 7.43)	4.52 (3.86, 6.38)	6.63 (5.07, 8.65)	<0.001
Neutrophil count, x10^9^/L	3.85 (2.70, 5.35)	2.75 (2.16, 3.87)	4.81 (3.66, 6.13)	<0.001
Lymphocyte count, x10^9^/L	1.31 (1.01, 1.74)	1.31 (1.10, 1.74)	1.26 (0.96, 1.74)	0.119
Monocyte count, x10^9^/L	0.38 (0.30, 0.51)	0.32 (0.26, 0.46)	0.44 (0.35, 0.58)	<0.001
Hemoglobin, g/L	124.00 (114.00, 134.00)	123.00 (114.50, 136.00)	124.00 (114.00, 133.00)	0.929
Platelet count, x10^9^/L	221.00 (176.00, 258.00)	200.00 (158.00, 234.50)	235.00 (199.00, 295.00)	<0.001
Alanine aminotransferase, U/L	16.00 (11.00, 25.00)	15.00 (11.50, 26.00)	16.00 (11.00, 22.00)	0.607
Aspartate aminotransferase, U/L	21.00 (16.00, 29.00)	20.50 (16.00, 30.00)	21.00 (17.00, 28.00)	0.336
Total bilirubin, µmol/L	10.30 (8.40, 13.00)	10.30 (8.40, 13.00)	10.40 (8.40, 13.10)	0.219
Albumin, g/L	36.50 (32.90, 39.40)	38.40 (33.40, 39.50)	35.40 (32.80, 38.60)	<0.001
Blood urea nitrogen, mmol/L	5.40 (4.20, 6.30)	5.60 (4.70, 6.55)	5.00 (4.10, 6.30)	<0.001
Creatinine, µmol/L	64.70 (54.20, 73.10)	65.50 (56.60, 73.20)	64.70 (53.20, 72.40)	0.532
Uric acid, µmol/L	299.70 (225.00, 354.70)	326.50 (254.40, 366.40)	291.60 (224.00, 345.90)	<0.001
Fasting blood glucose, mmol/L	5.13 (4.87, 5.54)	5.15 (4.89, 5.44)	5.11 (4.78, 5.70)	0.879
Triglycerides, mmol/L	1.20 (0.90, 1.77)	1.27 (0.95, 2.09)	1.15 (0.88, 1.49)	0.002
Total cholesterol, mmol/L	4.41 (3.89, 5.11)	4.41 (3.89, 5.11)	4.43 (3.70, 5.11)	0.450
High-density lipoprotein cholesterol, mmol/L	1.14 (0.97, 1.37)	1.08 (0.84, 1.29)	1.17 (1.01, 1.46)	<0.001
Low-density lipoprotein cholesterol, mmol/L	2.50 (1.99, 2.78)	2.60 (2.17, 3.14)	2.37 (1.92, 2.73)	<0.001
SII	665.83 (402.32, 959.64)	395.09 (318.22, 699.05)	851.58 (625.06, 1,119.83)	<0.001
Standardized SII	-0.24 (-0.65, 0.23)	-0.66 (-0.78, -0.18)	0.06 (-0.30, 0.48)	<0.001

SII, Systemic immune-inflammation index. Continuous variables were compared using the Mann-Whitney U test, and categorical variables were analyzed using the chi-square test or Fisher’s exact test.

**Table 2** categorized the 489 lung cancer patients into three groups based on SII levels: T1 (low SII, n = 159), T2 (moderate SII, n = 171), and T3 (high SII, n = 159). With increasing SII, patient age tended to decrease (P < 0.001), while the proportion of males rose significantly (54.1% in T3 vs. 39.6% in T1, P = 0.013). Higher SII levels were also associated with more frequent comorbidities, particularly diabetes (17.6% in T3 vs. 0% in T1, P < 0.001) and, to a lesser degree, hypertension (P = 0.020). Regarding metastatic patterns, patients in the T3 group had the highest rate of distant metastasis (55.3%) and the lowest rate of intrapulmonary metastasis (44.7%), differences that were statistically significant (P < 0.001). In addition, the T3 group exhibited lower BMI (P = 0.010), along with higher SBP and DBP (P = 0.001 and P = 0.013, respectively). Laboratory findings showed consistent trends: as SII increased, white blood cell, neutrophil, monocyte, and platelet counts rose markedly (all P < 0.001), while lymphocyte counts declined (P = 0.016), and hemoglobin levels were slightly higher in the T3 group (P = 0.041). For biochemical markers, BUN and uric acid were lower in the high SII group (both P ≤ 0.001), whereas FBG was elevated (P < 0.001). Similarly, triglyceride levels declined with higher SII (P < 0.001), while HDL-C increased (P < 0.001); no significant difference was observed in LDL-C (P = 0.057). In terms of outcomes, patients with higher SII faced a substantially greater risk of all-cause mortality, with 79.9% of T3 patients dying during follow-up compared to only 15.1% in T1 (P < 0.001).

**Table 2:** Baseline characteristics grouped by SII tertiles.

Characteristics	SII tertiles			P value
	T1	T2	T3	
N	159	171	159	
Age, years	68.00 (55.00, 74.00)	62.00 (53.00, 69.00)	62.00 (50.00, 67.00)	<0.001
Sex, n (%)				0.013
Male	63 (39.6%)	69 (40.4%)	86 (54.1%)	
Female	96 (60.4%)	102 (59.6%)	73 (45.9%)	
Smoking, n (%)	130 (81.8%)	144 (84.2%)	130 (81.8%)	0.793
Hypertension, n (%)	41 (25.8%)	32 (18.7%)	51 (32.1%)	0.020
Diabetes, n (%)	0 (0.0%)	14 (8.2%)	28 (17.6%)	<0.001
Adenocarcinoma, n (%)	151 (95.0%)	154 (90.1%)	139 (87.4%)	0.061
Staging, n (%)				0.518
Stage III	11 (6.9%)	14 (8.2%)	8 (5.0%)	
Stage IV	148 (93.1%)	157 (91.8%)	151 (95.0%)	
Site of metastasis, n (%)				<0.001
Intrapulmonary metastasis	100 (62.9%)	114 (66.7%)	71 (44.7%)	
Distant metastasis	59 (37.1%)	57 (33.3%)	88 (55.3%)	
Body mass index, kg/m^2^	22.64 (21.09, 25.95)	22.68 (20.03, 25.39)	22.32 (19.98, 25.59)	0.010
Systolic blood pressure, mmHg	126.00 (115.00, 130.00)	133.00 (116.00, 141.00)	130.00 (120.00, 138.00)	0.001
Diastolic blood pressure, mmHg	78.00 (70.00, 84.00)	82.00 (70.00, 88.00)	80.00 (72.00, 87.00)	0.013
White blood cell count, x10^9^/L	4.36 (3.75, 4.89)	5.67 (4.99, 7.56)	7.43 (6.06, 10.07)	<0.001
Neutrophil count, x10^9^/L	2.32 (2.07, 2.88)	4.07 (3.22, 5.33)	5.38 (4.71, 7.75)	<0.001
Lymphocyte count, x10^9^/L	1.31 (1.14, 1.74)	1.38 (1.01, 1.76)	1.14 (0.86, 1.73)	0.016
Monocyte count, x10^9^/L	0.31 (0.27, 0.46)	0.42 (0.29, 0.51)	0.45 (0.37, 0.63)	<0.001
Hemoglobin, g/L	123.00 (114.00, 135.00)	124.00 (116.00, 132.00)	127.00 (114.00, 139.00)	0.041
Platelet count, x10^9^/L	183.00 (156.00, 218.00)	229.00 (151.00, 248.00)	254.00 (233.00, 362.00)	<0.001
Alanine aminotransferase, U/L	16.00 (13.00, 31.00)	14.00 (11.00, 22.00)	15.00 (11.00, 23.00)	0.180
Aspartate aminotransferase, U/L	22.00 (16.00, 31.00)	21.00 (19.00, 27.00)	18.00 (14.00, 26.00)	0.002
Total bilirubin, µmol/L	9.70 (6.70, 11.50)	10.40 (9.50, 13.30)	10.40 (8.40, 14.20)	<0.001
Albumin, g/L	35.30 (32.80, 39.40)	36.60 (34.70, 39.00)	36.50 (32.80, 39.60)	0.658
Blood urea nitrogen, mmol/L	5.50 (4.70, 6.90)	5.40 (4.40, 6.30)	5.00 (3.90, 6.30)	<0.001
Creatinine, µmol/L	65.50 (56.60, 75.10)	62.10 (54.20, 73.10)	64.70 (53.10, 71.90)	0.127
Uric acid, µmol/L	324.70 (273.70, 354.70)	292.70 (224.20, 349.80)	282.00 (220.20, 360.80)	0.001
Fasting blood glucose, mmol/L	5.13 (4.89, 5.27)	5.10 (4.78, 5.48)	5.30 (4.91, 6.31)	<0.001
Triglycerides, mmol/L	1.52 (1.15, 2.19)	1.04 (0.77, 1.56)	1.11 (0.81, 1.44)	<0.001
Total cholesterol, mmol/L	4.41 (4.28, 5.09)	4.42 (3.74, 5.07)	4.43 (3.75, 5.24)	0.204
High-density lipoprotein cholesterol, mmol/L	1.08 (0.84, 1.33)	1.18 (1.00, 1.46)	1.16 (0.90, 1.29)	<0.001
Low-density lipoprotein cholesterol, mmol/L	2.58 (2.08, 3.03)	2.40 (1.95, 2.67)	2.51 (2.06, 2.76)	0.057
All-cause mortality				<0.001
No	135 (84.9%)	53 (31.0%)	32 (20.1%)	
Yes	24 (15.1%)	118 (69.0%)	127 (79.9%)	

SII, Systemic immune-inflammation index; T1, tertile 1; T2, tertile 2; T3, tertile 3. Continuous variables were compared using the Kruskal-Wallis test, and categorical variables were analyzed using the chi-square test or Fisher’s exact test.

### 3.2 Association between SII and all-cause mortality

**Figure 1:**
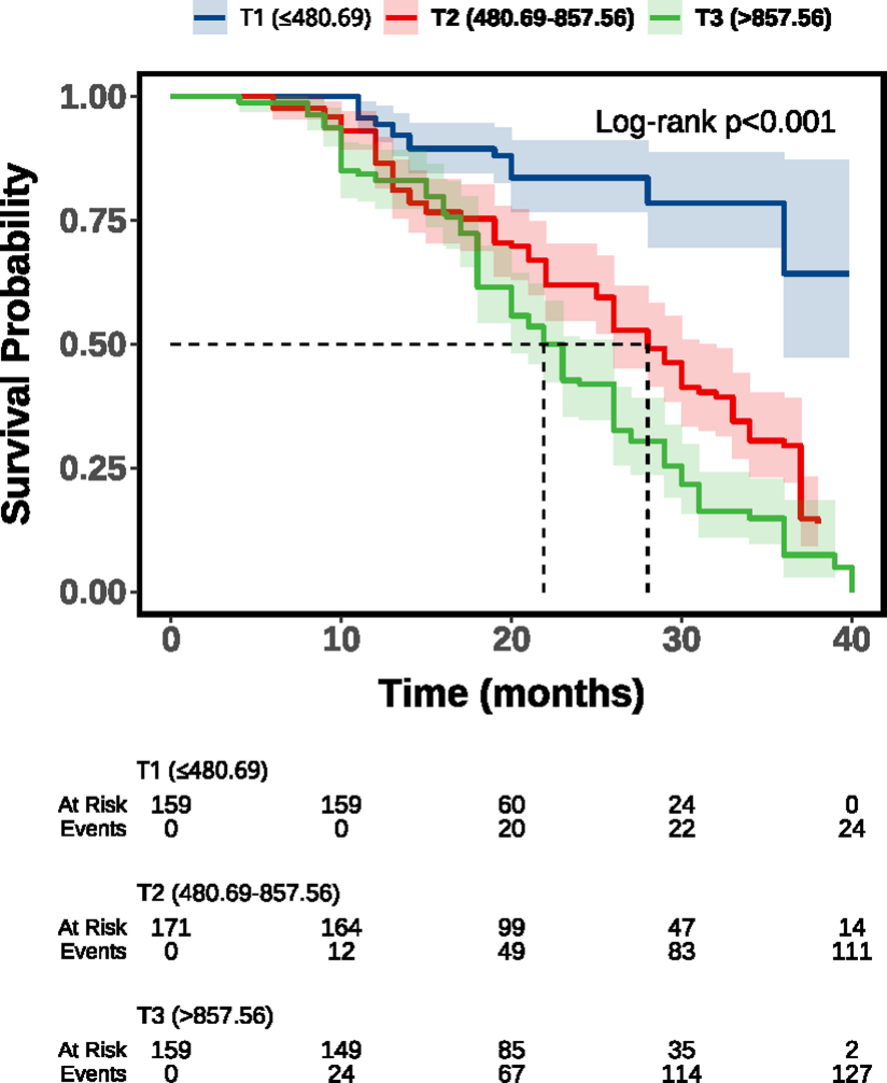
Kaplan–Meier survival curve of SII and all-cause mortality. SII, Systemic immune-inflammation index; T1, tertile 1; T2, tertile 2; T3, tertile 3.

The Kaplan–Meier survival curves in **Figure 1** demonstrated significant differences in survival across SII groups. Patients in the high SII group (T3) showed the steepest decline in survival over time, whereas those in the low SII group (T1) had the most favorable outcomes, with the intermediate group (T2) falling in between. The log-rank test confirmed that these differences were statistically significant (P < 0.001).

Univariable Cox regression analyses (**Table 3**) identified several factors associated with all-cause mortality, including male sex, hypertension, diabetes, histological subtype (adenocarcinoma as protective), stage IV disease, higher SBP and DBP, elevated white blood cell, neutrophil, monocyte, and platelet counts, as well as increased creatinine, FBG, and SII (HR = 1.000, P < 0.001) and standardized SII (HR = 1.350, P < 0.001). LDL-C was also inversely associated with mortality risk.

**Table 3:** Univariable Cox regression analysis of factors associated with the risk of all-cause mortality.

Characteristics	HR	95% CI	P value
Age	1.010	0.999, 1.020	0.070
Male	1.288	1.006, 1.651	0.045
Smoking	0.988	0.710, 1.375	0.943
Hypertension	1.443	1.107, 1.882	0.007
Diabetes	2.776	1.900, 4.054	<0.001
Adenocarcinoma	0.281	0.185, 0.426	<0.001
Staging			
Stage III	—	—	
Stage IV	2.714	1.578, 4.670	<0.001
Site of metastasis			
Intrapulmonary metastasis	—	—	
Distant metastasis	1.117	0.876, 1.424	0.373
Body mass index	1.029	0.992, 1.067	0.125
Systolic blood pressure	1.014	1.008, 1.021	<0.001
Diastolic blood pressure	1.014	1.004, 1.025	0.004
White blood cell count	1.146	1.094, 1.201	<0.001
Neutrophil count	1.167	1.105, 1.232	<0.001
Lymphocyte count	1.137	0.892, 1.450	0.298
Monocyte count	3.956	2.359, 6.632	<0.001
Hemoglobin	1.004	0.995, 1.012	0.399
Platelet count	1.003	1.002, 1.005	<0.001
Alanine aminotransferase	1.003	0.998, 1.009	0.197
Aspartate aminotransferase	0.998	0.988, 1.009	0.765
Total bilirubin	1.015	0.990, 1.041	0.232
Albumin	0.977	0.949, 1.006	0.126
Blood urea nitrogen	0.947	0.886, 1.011	0.105
Creatinine	1.009	1.003, 1.015	0.003
Uric acid	1.001	1.000, 1.002	0.304
Fasting blood glucose	1.306	1.169, 1.460	<0.001
Triglycerides	0.871	0.742, 1.021	0.089
Total cholesterol	0.929	0.801, 1.077	0.328
High-density lipoprotein cholesterol	0.861	0.593, 1.251	0.433
Low-density lipoprotein cholesterol	0.814	0.668, 0.992	0.041
SII	1.000	1.000, 1.001	<0.001
Standardized SII	1.350	1.233, 1.477	<0.001

SII, Systemic immune-inflammation index; HR, hazard ratio; CI, confidence interval.

Multivariable Cox regression analyses (**Table 4**) further confirmed the prognostic significance of SII. Whether treated as a continuous variable or stratified into tertiles, higher SII values were consistently linked to greater mortality risk. For standardized SII, HRs remained significant across all three models (HRs: 1.354, 1.329, and 1.222; all P < 0.001). In categorical analysis, compared with the T1 group, both T2 and T3 groups showed progressively increased risks of death. In the fully adjusted model (Model 3), HRs were 2.583 (95% CI: 1.630–4.095) for T2 and 4.475 (95% CI: 2.841–7.047) for T3, with a significant trend across tertiles (P for trend < 0.001).

**Table 4:** Multivariable association of SII with all-cause mortality.

Characteristics	Model 1			Model 2			Model 3		
	HR	95% CI	P value	HR	95% CI	P value	HR	95% CI	P value
As a continuous variable									
SII	1.000	1.000, 1.001	<0.001	1.000	1.000, 1.001	<0.001	1.000	1.000, 1.000	<0.001
Standardized SII	1.354	1.241, 1.477	<0.001	1.329	1.217, 1.452	<0.001	1.222	1.108, 1.347	<0.001
As a categorical variable									
T1	—	—		—	—		—	—	
T2	3.132	1.997, 4.912	<0.001	3.010	1.922, 4.713	<0.001	2.583	1.630, 4.095	<0.001
T3	5.437	3.476, 8.504	<0.001	4.834	3.093, 7.553	<0.001	4.475	2.841, 7.047	<0.001
P for trend			<0.001			<0.001			<0.001

Model 1: adjusted for age and sex; Model 2: adjusted for age, sex, hypertension, diabetes, adenocarcinoma, and staging; Model 3: adjusted for age, sex, hypertension, diabetes, adenocarcinoma, staging, systolic blood pressure, diastolic blood pressure, creatinine, fasting blood glucose, triglycerides, and low-density lipoprotein cholesterol.

SII, Systemic immune-inflammation index; T1, tertile 1; T2, tertile 2; T3, tertile 3; HR, hazard ratio; CI, confidence interval.

### 3.3 Subgroup analysis stratified by age and sex

After multivariable adjustment, subgroup analyses indicated that the association between SII and all-cause mortality varied across age and sex categories (**Table 5**). Among patients aged ≤ 60 years, both the T2 group (HR = 7.578, 95% CI: 2.229–25.763, P = 0.001) and the T3 group (HR = 30.727, 95% CI: 8.934–105.676, P < 0.001) showed significantly higher risks compared with T1, and standardized SII was also strongly associated with increased mortality (HR = 3.353, 95% CI: 2.580–4.357, P < 0.001). In contrast, among patients older than 60 years, only the T3 group was significantly associated with mortality (HR = 2.411, 95% CI: 1.305–4.451, P = 0.005), while neither the T2 group (HR = 1.737, 95% CI: 0.943–3.200, P = 0.077) nor standardized SII (HR = 1.045, 95% CI: 0.907–1.203, P = 0.544) reached statistical significance.

**Table 5:** Multivariable subgroup analysis of SII with all-cause mortality.

Subgroups	SII (T2 vs. T1)			SII (T3 vs. T1)			Standardized SII		
	HR	95% CI	P value	HR	95% CI	P value	HR	95% CI	P value
Age									
≤ 60 years	7.578	2.229, 25.763	0.001	30.727	8.934, 105.676	<0.001	3.353	2.580, 4.357	<0.001
> 60 years	1.737	0.943, 3.200	0.077	2.411	1.305, 4.451	0.005	1.045	0.907, 1.203	0.544
Sex									
Male	3.977	1.559, 10.146	0.004	4.744	1.900, 11.844	0.001	1.157	1.018, 1.316	0.026
Female	2.747	1.615, 4.674	<0.001	4.662	2.723, 7.981	<0.001	1.305	1.118, 1.524	0.001

Multivariable subgroup analysis adjusted for age, sex, hypertension, diabetes, adenocarcinoma, staging, systolic blood pressure, diastolic blood pressure, creatinine, fasting blood glucose, triglycerides, and low-density lipoprotein cholesterol.

SII, Systemic immune-inflammation index; T1, tertile 1; T2, tertile 2; T3, tertile 3; HR, hazard ratio; CI, confidence interval.

When stratified by sex, both men and women exhibited higher risks with elevated SII. In men, the T2 (HR = 3.977, 95% CI: 1.559–10.146, P = 0.004) and T3 groups (HR = 4.744, 95% CI: 1.900–11.844, P = 0.001) had significantly greater risks than T1, with standardized SII also showing significance (HR = 1.157, 95% CI: 1.018–1.316, P = 0.026). Similarly, in women, both the T2 (HR = 2.747, 95% CI: 1.615–4.674, P < 0.001) and T3 groups (HR = 4.662, 95% CI: 2.723–7.981, P < 0.001) were associated with higher risks compared to T1, and standardized SII consistently demonstrated a significant positive relationship (HR = 1.305, 95% CI: 1.118–1.524, P = 0.001).

### 3.4 Predictive value and dose-response relationship of SII for all-cause mortality

**Figure 2:**
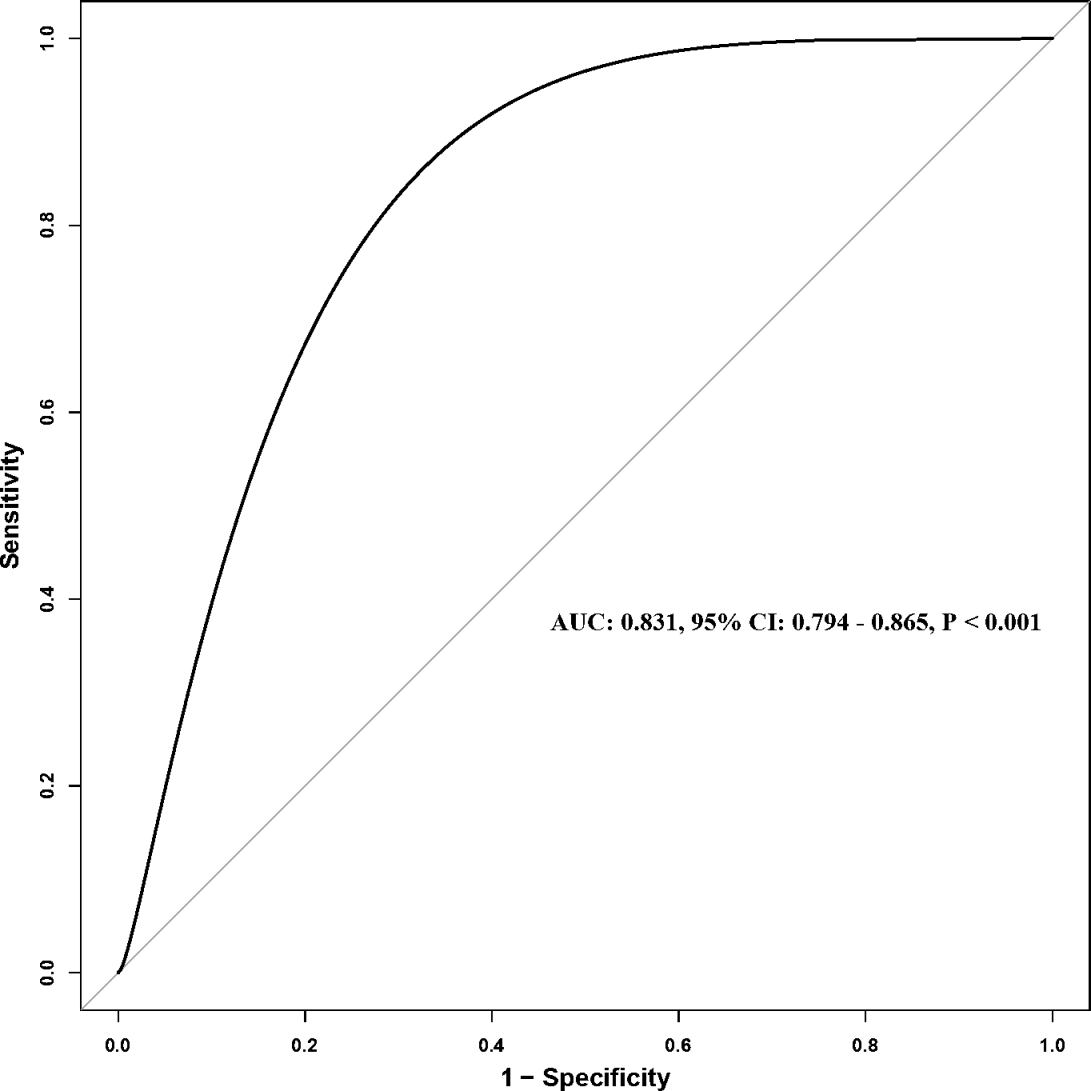
ROC curve of SII for predicting all-cause mortality. ROC, Receiver operating characteristic; SII, Systemic immune-inflammation index; AUC, Area under the curve; CI, Confidence interval.

**Figure 2** showed the ROC curve of SII for predicting all-cause mortality, with an AUC of 0.831 (95% CI: 0.794–0.865, P < 0.001), suggesting that SII has a strong discriminative ability for patient prognosis.

**Figure 3:**
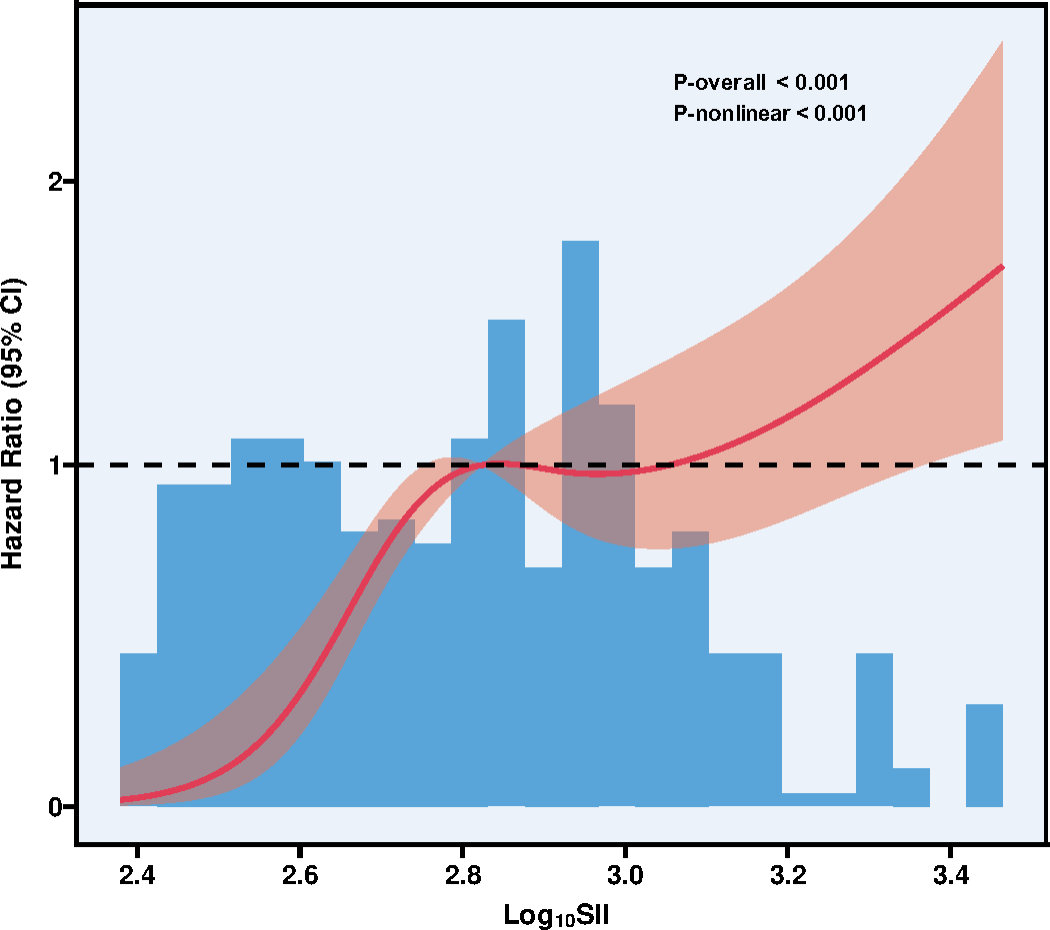
RCS analysis of SII and all-cause mortality. RCS, Restricted cubic spline; SII, Systemic immune-inflammation index; CI, Confidence interval.

**Figure 3** illustrated the results of the RCS analysis, which revealed a significant overall association between SII levels and the risk of all-cause mortality (P-overall < 0.001). Furthermore, the relationship was characterized by a non-linear pattern (P-nonlinear < 0.001).

## 4 Discussion

This study focused on lung cancer patients undergoing radiotherapy and systematically evaluated the relationship between SII and the risk of all-cause mortality. The findings demonstrated that SII not only distinguished survival outcomes among patients but also remained an independent adverse prognostic factor after multivariable adjustment. Higher SII levels were associated with a greater prevalence of metabolic comorbidities, marked increases in inflammatory cell counts, and ultimately poorer survival. Both the ROC curve analysis and the RCS model further indicated that SII had strong predictive capacity for prognosis, with a significant nonlinear dose–response pattern. Specifically, the RCS analysis suggested that the association between SII and all-cause mortality was not linear but followed a complex trend. The risk of mortality increased gradually at lower SII levels, remained relatively stable within a moderate range, and then increased more noticeably at higher SII levels. Notably, some intermediate ranges showed wider confidence intervals that crossed unity, indicating greater statistical uncertainty and warranting cautious interpretation. In contrast, at higher SII levels, the upward trend became more apparent, suggesting a potentially more clinically meaningful increase in risk. This non-linear relationship has important clinical implications. It indicates that the effect of SII on mortality risk may vary across different ranges rather than increase uniformly. While moderate elevations in SII may correspond to limited changes in risk, substantially elevated SII levels may be associated with a more pronounced increase in mortality risk. Therefore, clinicians should avoid assuming a purely linear relationship and instead pay particular attention to patients with markedly elevated SII, who may represent a high-risk population requiring closer monitoring and more intensive management. Furthermore, these findings highlight the need for future studies to further explore potential thresholds or high-risk ranges of SII in larger, multicenter prospective cohorts, which may help refine risk stratification and guide individualized clinical interventions. Taken together, these results suggest that SII, as a simple, cost-effective, and reproducible marker of systemic inflammation and immunity, holds promise as an important prognostic tool for patients with lung cancer receiving radiotherapy. Clinically, it may assist physicians in rapidly identifying high-risk individuals based on routine blood tests, thereby facilitating personalized treatment planning and follow-up management. In this context, a deeper understanding of the role and development of SII in oncology research is particularly valuable.

In recent years, the SII, which reflects the balance between host inflammatory responses and immune status, has attracted considerable attention in prognostic studies of lung cancer. Multiple meta-analyses and systematic reviews have consistently shown that elevated SII levels are closely linked to unfavorable outcomes in patients with non-small cell lung cancer (NSCLC). For example, Huang et al. (17), in a meta-analysis of 17 studies involving 8,877 patients, demonstrated that high SII was associated with significantly shorter overall survival and progression-free survival (PFS), and correlated with more advanced tumor stage, suggesting that SII not only predicts prognosis but also mirrors tumor progression biology (17). Among patients treated with immune checkpoint inhibitors (ICIs), Yang et al. (18) and Zhang et al. (13), analyzing 20 and 13 studies respectively, reported that high pretreatment SII was significantly associated with poorer OS and PFS, with the effect particularly pronounced in patients receiving ICIs alone rather than in combination with chemotherapy (13, 18). These findings highlight the potential of SII as a simple hematological marker to predict immunotherapy efficacy. Beyond NSCLC, SII has also shown prognostic value in small-cell lung cancer (SCLC). Zhou et al. (19), in a pooled analysis of 2,267 cases, found that high SII was strongly associated with worse OS, particularly in extensive-stage disease (19). More recently, Hamakawa et al. (20) confirmed that elevated SII independently predicted shorter OS and PFS in SCLC patients undergoing ICI maintenance therapy, reinforcing its utility in the immunotherapy setting (20). Similarly, Fu et al. (21), in a large cohort of 3,984 NSCLC patients, reported that the prognostic significance of SII was especially evident in stage I disease, adenocarcinoma, and solid nodules, providing important guidance for identifying subgroups where SII may be most clinically useful (21). Other investigations have extended the scope of SII to molecularly defined populations and dynamic monitoring. Chen et al. (14) observed that low pre-radiotherapy SII predicted longer OS in advanced EGFR-mutant lung adenocarcinoma, independent of other clinical variables (14). Huang et al. (22) emphasized that changes in SII (ΔSII) before and after concurrent chemoradiotherapy had greater prognostic accuracy than a single measurement, serving as an independent predictor of OS and PFS (22). Likewise, Zhang et al. (23) demonstrated that high SII independently predicted worse OS and PFS in NSCLC patients with brain metastases treated with stereotactic radiotherapy (23). Importantly, SII has also been explored in contexts beyond survival prediction. Zhang et al. (24) reported that high SII was significantly associated with venous thromboembolism (VTE) in lung cancer patients, and could be integrated into a nomogram to improve VTE risk stratification (24). Mao et al. (25) further found that SII, together with pulmonary function parameters, was a strong predictor of postoperative pulmonary complications after lung cancer resection, underscoring its potential utility across perioperative management (25). Taken together, the current body of evidence strongly supports SII as a versatile biomarker in lung cancer, spanning NSCLC and SCLC, surgery, radiotherapy, chemoradiotherapy, immunotherapy, and even complication prediction. Nevertheless, most prior studies have concentrated on surgical or immunotherapy cohorts, with relatively few focusing specifically on patients undergoing radiotherapy. Against this background, the present study offered two distinct advantages. First, it included a relatively large cohort of 489 patients who received standardized radiotherapy at a single center with complete follow-up, providing robust and representative data. Second, beyond establishing the association between baseline SII and all-cause mortality, this study applied stratified analyses, ROC curves, and RCS models to reveal the non-linear dose–response relationship, thereby offering a more nuanced assessment of the prognostic role of SII. Collectively, these strengths enable our study to fill an important gap in the literature and highlight SII as a convenient, cost-effective, and reproducible biomarker that may support individualized decision-making in the radiotherapy setting.

From a biological perspective, an elevated SII reflects a dual condition of intensified inflammation and weakened immune defense. Neutrophils can release proinflammatory cytokines and reactive oxygen species that promote tumor proliferation, angiogenesis, and metastasis, while also suppressing T-cell activity and thereby diminishing antitumor immunity (26). Platelets, beyond their role in hemostasis and coagulation, can interact with circulating tumor cells, shielding them from immune surveillance and facilitating the establishment of metastatic niches (27). In contrast, lymphocytes are central to adaptive immunity, contributing to tumor recognition and elimination; a reduced lymphocyte count often indicates impaired immune competence (28). Thus, a high SII, characterized by elevated neutrophil and platelet counts alongside decreased lymphocyte levels, represents a state of “inflammation-driven and immunity-suppressed” physiology. In the context of radiotherapy, this imbalance may be further amplified, as radiation induces inflammatory responses while transiently dampening immune function. Patients with high SII are therefore more susceptible to tumor progression and recurrence during treatment, ultimately leading to worse outcomes. The findings of this study are consistent with these mechanistic insights, providing a biological rationale for the observed clinical significance.

Despite the clinically meaningful findings of this study, several limitations should be acknowledged. First, as a single-center retrospective design, the study was subject to potential selection and information bias, and the conclusions warrant confirmation in multicenter prospective cohorts. Second, SII is a dynamic marker influenced by multiple factors such as infection, medication use, and acute inflammatory responses. Because the present analysis relied only on a single baseline measurement prior to radiotherapy, it may not capture temporal variations. Third, although multiple clinical and laboratory variables were included in the multivariable models, residual confounding cannot be excluded. Patient-specific factors such as genetic background, molecular subtype, and details of radiotherapy regimens may also contribute to outcomes. In particular, information on whether patients received chemotherapy or immunotherapy, as well as details regarding radiotherapy dose intensity and number of sessions, was incompletely recorded in the electronic medical records and therefore could not be included in the analysis. This limitation reflects the retrospective nature of the study and may have influenced treatment intensity and outcomes, especially in older patients or those with multiple comorbidities. Future studies should aim to systematically collect comprehensive treatment data in multicenter prospective cohorts to better account for these factors and validate the prognostic value of SII. Finally, this study focused exclusively on all-cause mortality and did not examine cancer-specific mortality or progression-free survival, which limits the scope of interpretation. These constraints highlight the need for future investigations with larger populations, longitudinal monitoring, and integration of multidimensional data to further validate and refine the prognostic value of SII.

## 5 Conclusion

In conclusion, this study demonstrates that SII serves as an independent predictor of all-cause mortality among lung cancer patients receiving radiotherapy and can provide additional prognostic information beyond routine hematological assessments. Clinically, SII represents a simple, inexpensive, and reproducible tool for risk stratification, which may assist clinicians in refining individualized treatment plans and follow-up strategies. In this context, patients with markedly elevated SII may be considered at higher risk and could benefit from closer surveillance, more frequent follow-up, and more attentive monitoring of disease progression. Furthermore, integrating SII into clinical decision-making may help inform individualized management approaches, including optimization of supportive care and multidisciplinary evaluation of treatment strategies. However, these potential applications should be interpreted with caution, as they are derived from observational findings and require further validation in prospective studies. Future work should focus on large-scale, multicenter prospective studies and incorporate molecular biomarkers, imaging parameters, and radiotherapy dosimetric characteristics to further elucidate the interactions between SII and lung cancer outcomes, thereby facilitating its standardized implementation in clinical practice.

## Data Availability

The raw data supporting the conclusions of this article will be made available by the authors, without undue reservation.
